# Supplementary material for: Heterogeneity of Amino Acid Profiles of Proneural and Mesenchymal Brain-Tumor Initiating Cells
Source: Int J Mol Sci. 2023 Feb 6;24(4):3199. doi: 10.3390/ijms24043199 (PMC9962848; doi:10.3390/ijms24043199)
Supplement: Supplementary file 1 [file ijms-24-03199-s001.zip › ijms-2169466-supplementary.pdf]

Supplementary Material

Suppl. Table S1: Hallmark hypoxia I

Gene symbol	Gene title	Rank in gene list	Rank metric score	Core enrichment	
BCAN	brevican	0	4.877.999.782.562.250	No	Proneural
null	null	9	3.808.000.087.738.030	No	Proneural
KIF5A	kinesin family member 5A	212	14.859.999.418.258.600	No	Proneural
MAP3K1	mitogen-activated protein kinase kinase kinase 1	223	1.437.999.963.760.370	No	Proneural
SDC3	syndecan 3 (N-syndecan)	330	10.670.000.314.712.500	No	Proneural
EFNA3	ephrin-A3	383	0.963999987	No	Proneural
HDLBP	high density lipoprotein binding protein (vigilin)	557	-0.330000013	No	Mesenchymal
PRDX5	peroxiredoxin 5	633	-0.675999999	No	Mesenchymal
GBE1	glucan (1,4-alpha-), branching enzyme 1 (glycogen branching enzyme, Andersen disease, glycogen storage disease type IV)	768	-0.999000013	No	Mesenchymal
TNFAIP3	tumor necrosis factor, alpha-induced protein 3	839	-12.450.000.047.683.700	No	Mesenchymal
GLRX	glutaredoxin (thioltransferase)	899	-1.440.999.984.741.210	Yes	Mesenchymal
MYH9	myosin, heavy chain 9, non-muscle	904	-14.639.999.866.485.500	Yes	Mesenchymal
AMPD3	adenosine monophosphate deaminase (isoform E)	934	-15.820.000.171.661.300	Yes	Mesenchymal
COL5A1	collagen, type V, alpha 1	956	-1.656.000.018.119.810	Yes	Mesenchymal
SLC6A6	solute carrier family 6 (neurotransmitter transporter, taurine), member 6	958	-1.659.999.966.621.390	Yes	Mesenchymal
TMEM45A	transmembrane protein 45A	1009	-18.389.999.866.485.500	Yes	Mesenchymal
ANXA2	annexin A2	1016	-18.619.999.885.559.000	Yes	Mesenchymal
ERRFI1	ERBB receptor feedback inhibitor 1	1029	-1.899.999.976.158.140	Yes	Mesenchymal
FOSL2	FOS-like antigen 2	1046	-19.989.999.532.699.500	Yes	Mesenchymal
SDC4	syndecan 4 (amphiglycan, ryudocan)	1069	-211.899.995.803.833	Yes	Mesenchymal
P4HA2	procollagen-proline, 2-oxoglutarate 4-dioxygenase (proline 4-hydroxylase), alpha polypeptide II	1076	-21.579.999.923.706.000	Yes	Mesenchymal

Suppl. Table S1: Hallmark hypoxia cont.

Gene symbol	Gene title	Rank in gene list	Rank metric score	Core enrichment	
IL6	interleukin 6 (interferon, beta 2)	1080	-21.730.000.972.747.800	Yes	Mesenchymal
TGM2	transglutaminase 2 (C polypeptide, protein-glutamine-gamma-glutamyltransferase)	1096	-2.249.000.072.479.240	Yes	Mesenchymal
IER3	immediate early response 3	1124	-23.499.999.046.325.600	Yes	Mesenchymal
PTRF	polymerase I and transcript release factor	1126	-236.899.995.803.833	Yes	Mesenchymal
CYR61	cysteine-rich, angiogenic inducer, 61	1145	-24.830.000.400.543.200	Yes	Mesenchymal
SERPINE1	serpin peptidase inhibitor, clade E (nexin, plasminogen activator inhibitor type 1), member 1	1156	-25.899.999.141.693.100	Yes	Mesenchymal
CTGF	connective tissue growth factor	1173	-27.149.999.141.693.100	Yes	Mesenchymal
PLAUR	plasminogen activator, urokinase receptor	1179	-27.750.000.953.674.300	Yes	Mesenchymal
LOX	lysyl oxidase	1209	-32.049.999.237.060.500	Yes	Mesenchymal
TPBG	trophoblast glycoprotein	1223	-3.431.999.921.798.700	Yes	Mesenchymal
ADM	adrenomedullin	1231	-36.470.000.743.865.900	Yes	Mesenchymal
CAV1	caveolin 1, caveolae protein, 22kDa	1245	-4.706.999.778.747.550	Yes	Mesenchymal
TGFBI	transforming growth factor, beta-induced, 68kDa	1247	-5.078.999.996.185.300	Yes	Mesenchymal

Suppl. Table S2: Lipid metabolic process

Gene symbol	Gene title	Rank in gene list	Rank metric score	Core enrichment	
ABCD2	ATP-binding cassette, sub-family D (ALD), member 2	101	2.125	No	Proneural
HDLBP	high density lipoprotein binding protein (vigilin)	557	-0.33000001311302185	No	Mesenchymal
CDS1	CDP-diacylglycerol synthase (phosphatidate cytidylyltransferase) 1	619	-0.6240000128746033	No	Mesenchymal
TNFRSF1A	tumor necrosis factor receptor superfamily, member 1A	699	-0.8159999847412109	No	Mesenchymal
NPC2	Niemann-Pick disease, type C2	766	-0.9940000176429749	No	Mesenchymal
PLCD1	phospholipase C, delta 1	848	-12.799.999.713.897.700	Yes	Mesenchymal
LDLR	low density lipoprotein receptor (familial hypercholesterolemia)	859	-13.079.999.685.287.400	Yes	Mesenchymal
ABCB4	ATP-binding cassette, sub-family B (MDR/TAP), member 4	882	-13.919.999.599.456.700	Yes	Mesenchymal
CPT1A	carnitine palmitoyltransferase 1A (liver)	903	-14.620.000.123.977.600	Yes	Mesenchymal
AKR1B10	aldo-keto reductase family 1, member B10 (aldose reductase)	1043	-19.859.999.418.258.600	Yes	Mesenchymal
ALDH1A3	aldehyde dehydrogenase 1 family, member A3	1057	-2.063.999.891.281.120	Yes	Mesenchymal
null	null	1091	-22.300.000.190.734.800	Yes	Mesenchymal
APOL1	apolipoprotein L, 1	1095	-2.240.000.009.536.740	Yes	Mesenchymal
AKR1C1	aldo-keto reductase family 1, member C1 (dihydrodiol dehydrogenase 1; 20-alpha (3-alpha)-hydroxysteroid dehydrogenase)	1177	-2.752.000.093.460.080	Yes	Mesenchymal
CH25H	cholesterol 25-hydroxylase	1214	-3.259.999.990.463.250	Yes	Mesenchymal
ADM	adrenomedullin	1231	-36.470.000.743.865.900	Yes	Mesenchymal

Suppl. Table S3: Oxidoreductase activity

Gene symbol	Gene title	Rank in gene list	Rank metric score	Core enrichment	
GLDC	glycine dehydrogenase (decarboxylating)	69	25.269.999.504.089.300	No	Proneural
ME2	malic enzyme 2, NAD(+)-dependent, mitochondrial	416	0.8669999837875366	No	Proneural
PRDX2	peroxiredoxin 2	525	0.5799999833106995	No	Proneural
CAT	catalase	673	-0.765999972820282	No	Mesenchymal
NQO2	NAD(P)H dehydrogenase, quinone 2	695	-0.8080000281333923	No	Mesenchymal
H6PD	hexose-6-phosphate dehydrogenase (glucose 1-dehydrogenase)	803	-11.319.999.694.824.200	No	Mesenchymal
GLRX	glutaredoxin (thioltransferase)	899	-1.440.999.984.741.210	Yes	Mesenchymal
CYB5R2	cytochrome b5 reductase 2	936	-15.829.999.446.868.800	Yes	Mesenchymal
VCL	vinculin	989	-17.680.000.066.757.200	Yes	Mesenchymal
CYBRD1	cytochrome b reductase 1	1005	-1.815.999.984.741.210	Yes	Mesenchymal
DIO2	deiodinase, iodothyronine, type II	1006	-18.259.999.752.044.600	Yes	Mesenchymal
NQO1	NAD(P)H dehydrogenase, quinone 1	1008	-18.289.999.961.853.000	Yes	Mesenchymal
AKR1B10	aldo-keto reductase family 1, member B10 (aldose reductase)	1043	-19.859.999.418.258.600	Yes	Mesenchymal
ALDH1A3	aldehyde dehydrogenase 1 family, member A3	1057	-2.063.999.891.281.120	Yes	Mesenchymal
P4HA2	procollagen-proline, 2-oxoglutarate 4-dioxygenase (proline 4-hydroxylase), alpha polypeptide II	1076	-21.579.999.923.706.000	Yes	Mesenchymal
ALDH1A1	aldehyde dehydrogenase 1 family, member A1	1106	-22.860.000.133.514.400	Yes	Mesenchymal
MSRB3	methionine sulfoxide reductase B3	1137	-2.444.000.005.722.040	Yes	Mesenchymal
LOXL2	lysyl oxidase-like 2	1146	-2.496.999.979.019.160	Yes	Mesenchymal
AKR1C1	aldo-keto reductase family 1, member C1 (dihydrodiol dehydrogenase 1; 20-alpha (3-alpha)-hydroxysteroid dehydrogenase)	1177	-2.752.000.093.460.080	Yes	Mesenchymal
LOX	lysyl oxidase	1209	-32.049.999.237.060.500	Yes	Mesenchymal
CYP1B1	cytochrome P450, family 1, subfamily B, polypeptide 1	1246	-5.040.999.889.373.770	Yes	Mesenchymal

Supplementary figures

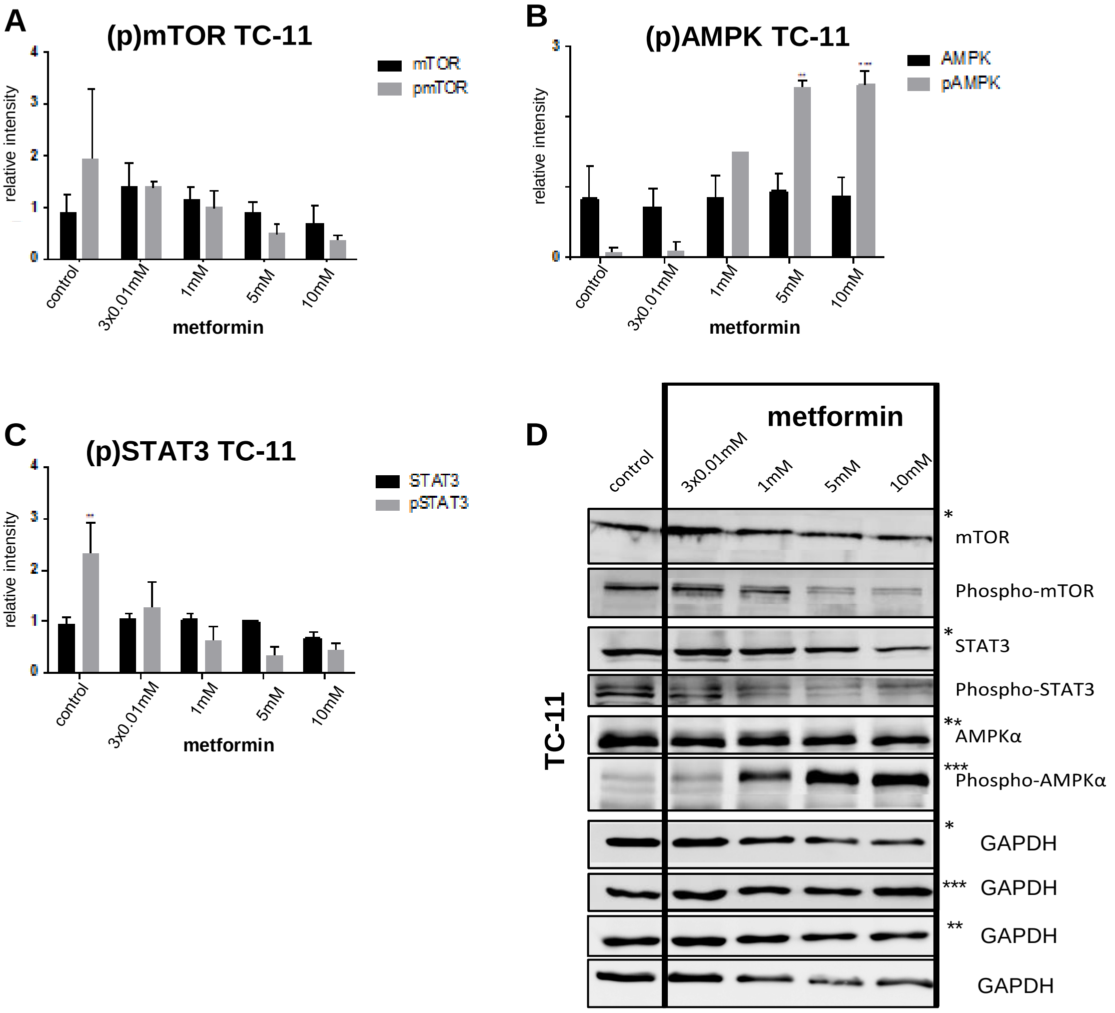

Supplementary Figure S1: Signaling in TC-11 and Western Blot quantification. Protein expression of **(A)** (p)mTOR, **(B)** (p)AMPK and **(C)** (p)STAT3 was explored after 48 h in TC-11. One representative Western Blot of TC-11 is depicted in **(D)**. Western blots were repeated three times with at least two biological replicates and quantified using Image J, version 1.49. Asterisks indicate * $p < 0.05$, ** $p < 0.01$, *** $p<0.001$.

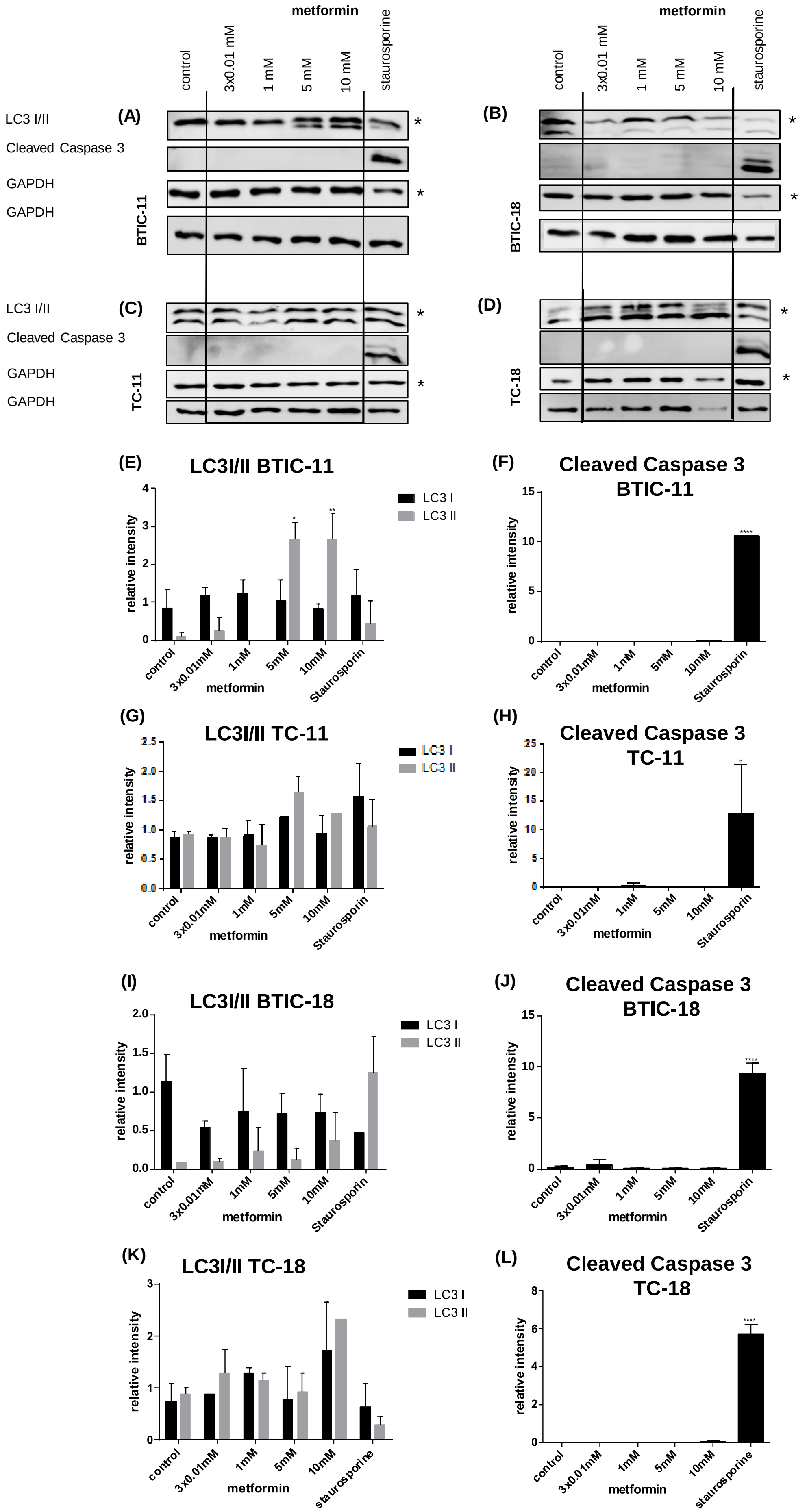

metformin
control
3x0.01 mM
1 mM
5 mM
10 mM
staurosporine
LC3 I/II
Cleaved Caspase 3
GAPDH
GAPDH
(A)
(B)
(C)
(D)
BTIC-11
BTIC-18
TC-11
TC-18
*
(E)
LC3I/II BTIC-11
(F)
Cleaved Caspase 3 BTIC-11
(G)
LC3I/II TC-11
(H)
Cleaved Caspase 3 TC-11
(I)
LC3I/II BTIC-18
(J)
Cleaved Caspase 3 BTIC-18
(K)
LC3I/II TC-18
(L)
Cleaved Caspase 3 TC-18
LC3 I
LC3 II
relative intensity
control
3x0.01mM
1mM
5mM
10mM
Staurosporin
staurosporine
metformin

Supplementary Figure S2: Western Blot analysis of autophagy and Caspase 3-dependent apoptosis in BTICs and TCs. Protein expression of LC3 I/II and cleaved caspase 3 of controls or cells treated with increasing doses of metformin as indicated was determined for **(A)** BTIC-11 **(B)** BTIC-18, **(C)** TC-11 and **(D)** TC-18. 1 µM staurosporin was used as positive control for induction of caspase-dependent apoptosis. Western blots were repeated three times with at least two biological replicates and quantified using Image J for LC3 I/II in BTIC-11 **(E)**, TC-11 **(G)**, BTIC-18 **(I)** and TC-18 **(K)** or cleaved caspase 3 in BTIC-11 **(F)**, TC-11 **(H)**, BTIC-18 **(J)** and TC-18 **(L)**. Asterisks indicate * $p < 0.05$, ** $p < 0.01$, **** $p < 0.001$.

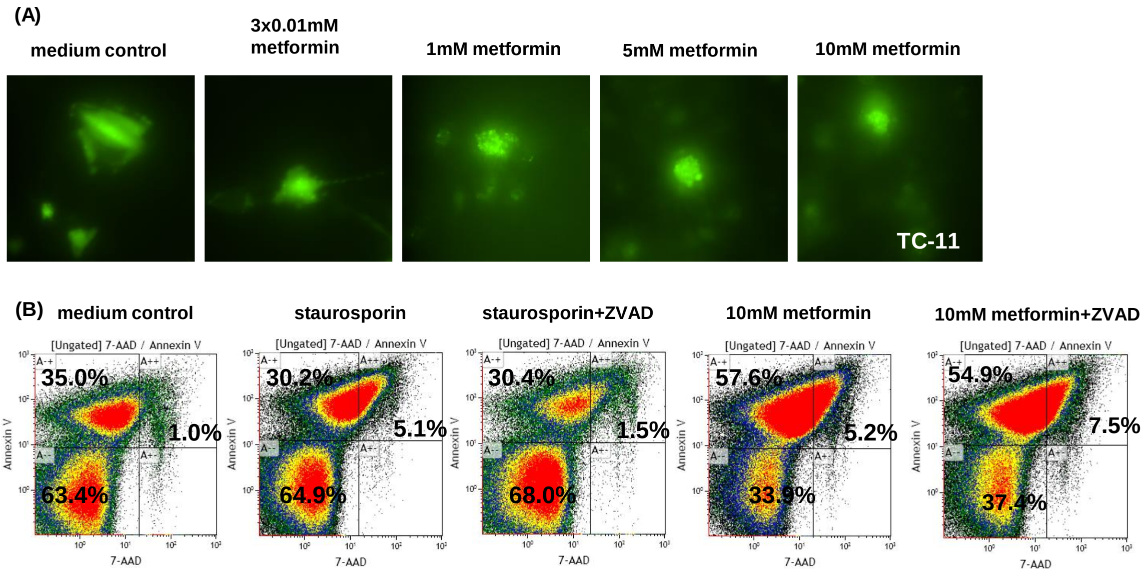

Supplementary Figure S3: (A) Live imaging of autophagosome formation was performed after transient transfection with an LC3-GFP-Plasmid over 48 h in TC-11. **(B)** Annexin/7-AAD FACS analysis showed increased apoptotic and necrotic cell death in glioma cells treated with staurosporine and metformin, which could be inhibited by treatment with the caspase-inhibitor ZVAD in glioma cells treated with staurosporine only but not in cells treated with metformin in addition.

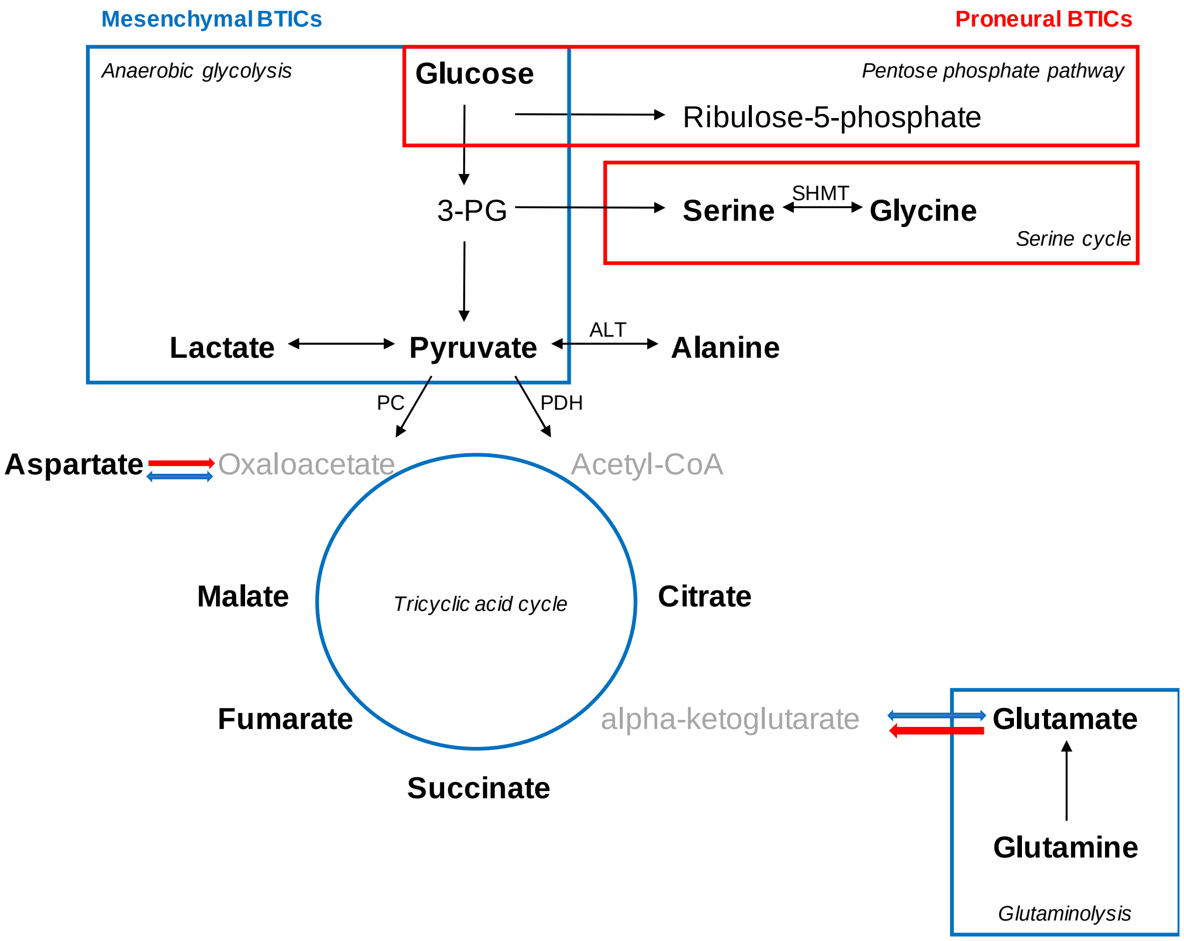

Supplementary Figure S4: Summary of metabolic pathways observed in the investigated proneural and mesenchymal BTICs. 3-PG= 3-phosphoglycerate, SHMT= serine hydroxymethyltransferase, ALT= alanine aminotransferase, PC=pyruvate carboxylase, PDH=pyruvate dehydrogenase.
